# G protein-coupled receptors: a gateway to targeting oncogenic EVs?

**DOI:** 10.20517/evcna.2024.10

**Published:** 2024-05-23

**Authors:** Lotte Di Niro, Amber C. Linders, Thomas Glynn, D. Michiel Pegtel, Marco Siderius, Caitrin Crudden, Martine J. Smit

**Affiliations:** ^1^Department of Chemistry and Pharmaceutical Sciences, Division of Medicinal Chemistry, Amsterdam Institute for Molecular and Life Sciences, Vrije Universiteit Amsterdam, Amsterdam 1081 HV, The Netherlands.; ^2^Department of Pathology, Cancer Center Amsterdam, Amsterdam UMC location Vrije Universiteit Amsterdam, Amsterdam 1081 HV, The Netherlands.

**Keywords:** G protein-coupled receptors (GPCRs), extracellular vesicles (EVs), oncogenic signaling, therapeutic targeting

## Abstract

Dysregulated intercellular communication is a key feature driving cancer progression. Recently, extracellular vesicles (EVs) have added a new channel to this dense communication network. Despite solid evidence that EVs are central mediators of dysregulated signaling in onco-pathological settings, this has yet to be translated into clinically actionable strategies. The heterogeneity of EV cargo molecules, plasticity of biogenesis routes, and large overlap with their role in physiological communication, complicate a potential targeting strategy. However, recent work has linked EV biology to perhaps the "most druggable" proteins - G protein-coupled receptors (GPCRs). GPCR targeting accounts for ~60% of drugs in development and more than a third of all currently approved drugs, spanning almost all areas of medicine. Although several GPCRs have been linked to cancer initiation and progression, relatively few agents have made it into oncological regimes, suggesting that their potential is underexploited. Herein, we examine the molecular mechanisms linking GPCRs to EV communication in cancer settings. We propose that GPCRs hold potential in the search for EV-targeting in oncology.

## INTRODUCTION

The notion that extracellular vesicles (EVs) drive tumor progression is backed up by ample evidence^[1,2]^. EVs have been implicated in several aspects of cancer, including tumor survival, angiogenesis, pre-metastatic niche formation, and drug resistance. EVs play an important role in intercellular communication through the transfer of the information contained in the vesicles to local or distant sites, maintaining the organism’s homeostasis. Tumors can hijack this network of communication, co-opting the EV machinery toward cargo selection and secretion of molecules that promote the establishment of a tumor-permissive local microenvironment. The EV-mediated crosstalk between cancer and stromal cells can, for example, suppress an antitumoral immune reaction^[3]^, establish a pre-metastatic niche^[4]^ or provide a means to expel targeting-agents^[5]^-mediating drug resistance.

Intercepting the EVs secreted from cancer cells has therefore often been proposed as a therapeutic strategy. This would be beneficial because EVs facilitate many cancer hallmarks^[3-5]^. However, developing a clinically feasible EV-targeting strategy has proven challenging. One might speculate that an approach to reduce the detrimental effects of cancer-released EVs could be to inhibit their biogenesis, secretion, or uptake by the recipient cells. Several studies have already shown that modulating certain proteins or lipids (e.g., Rab27a, nSMase2, farnesyl transferase, Ras, ceramide) involved in the biogenesis and secretion mechanisms of EVs effectively reduced the level of tumor EVs in the extracellular environment, thereby increasing sensitivity to chemotherapy and reducing tumor progression^[6-13]^. Unfortunately, none of these strategies have progressed beyond *in vivo* modeling.

Currently, the major hurdle is the development of drugs that selectively target tumor-derived EV biogenesis or secretion while sparing EV-mediated communication in physiological cell/tissue function^[14]^. It is not yet clear whether there are unique drivers of EV biogenesis in cancer cells or what these tumor-specific stimulants could be. With two remaining hurdles in mind - cancer specificity and druggability - attention is warranted to the emergence of studies linking EV biology to the lucrative family of G protein-coupled receptors (GPCRs). This large family of membrane receptors is involved in the regulation of a plethora of physiological and pathological processes^[15-22]^. In 2017, it was estimated that approximately 25-36% of all FDA- and EMA-approved drugs target a GPCR^[23,24]^. These receptors have an inherent druggability due to, for example, their favorable subcellular location, their dynamic structures, and their ability to activate multiple signaling pathways^[25]^. In addition, GPCRs have been identified as modulators of EV biogenesis, secretion, and uptake by recipient cells^[26]^. Concordantly, over the last few years, numerous studies have emerged regarding the GPCR contribution to EV-mediated cancer progression, which might be the targetable angle the EV community has been looking for. In this review, we provide an overview of how GPCR signaling and EV trafficking cooperate to support tumor survival and progression, and propose the potential for targeting GPCRs to modulate cancer-associated EVs as a therapeutic strategy.

## G PROTEIN-COUPLED RECEPTORS AND THEIR ONCOMODULATORY PROPERTIES

**Figure 1:**
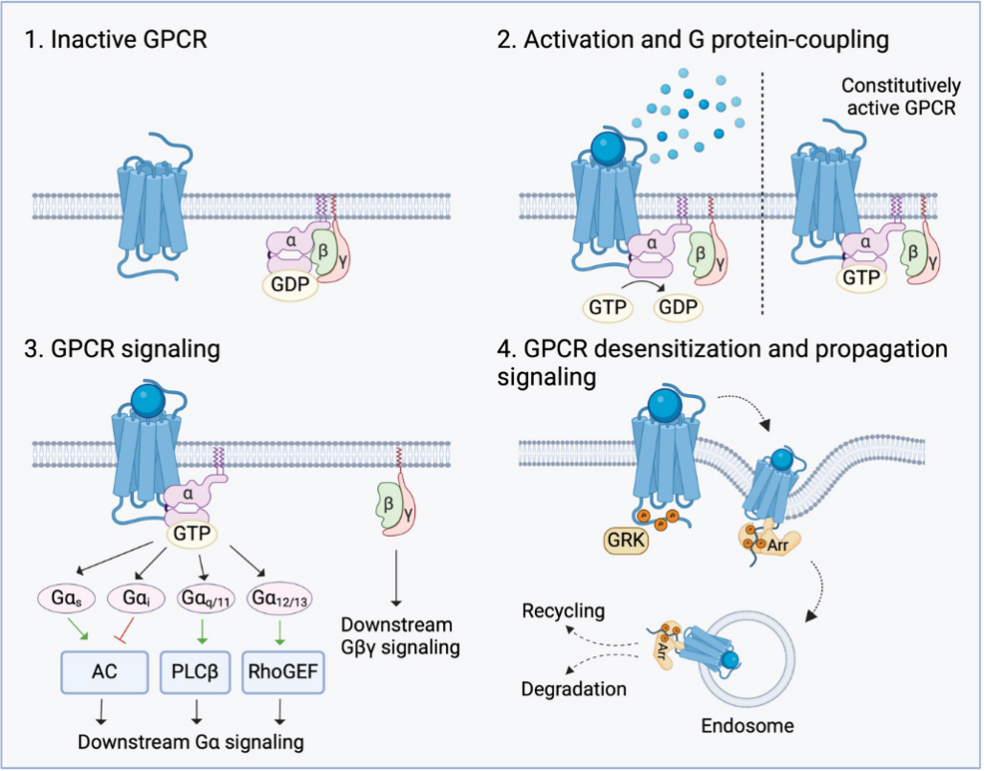
Schematic overview of GPCR signaling. (1) GPCR in its inactive state, where the heterotrimeric G protein complex, consisting of a Gα-subunit and a Gβγ-subunit, is still associated and GDP bound; (2) Ligand binding induces the GPCR to undergo a conformational change that initiates G protein coupling. The heterotrimeric G protein complex is activated by GDP exchange for GTP. For a constitutively active GPCR, the G protein constantly couples to the receptor; (3) Subsequently, the subunits dissociate and interact with their corresponding effector proteins, regulating downstream signaling; (4) GPCR signaling is downregulated by G protein-coupled receptor kinase (GRK)-mediated phosphorylation of the C-terminus. Subsequent arrestin binding initiates receptor endocytosis, which can lead to receptor degradation or recycling back to the plasma membrane, as well as endosomal signaling propagation. GPCR: G protein-coupled receptor; AC: adenylyl cyclase; PLCβ: phospholipase C-beta; GRK: G protein-coupled receptor kinase; Arr: arrestin; GDP: guanosine diphosphate; GTP: guanosine triphosphate.

GPCRs are the largest family of membrane-bound receptors (~ 800 members in humans)^[22]^. They are characterized by their seven transmembrane-spanning domains separated by alternating intracellular and extracellular loops. In humans, the GPCR family consists of four main classes based on their structural similarities: rhodopsin receptor-like (Class A), secretin and adhesion receptor-like (Class B), metabotropic glutamate receptor-like (Class C), and frizzled receptor-like (Class F)^[27]^. Depending on the type of GPCR, receptor activation is initiated upon ligand binding (including neurotransmitters, hormones, lipids, photons, and chemokines) to either an extracellular domain or a transmembrane domain, or a combination of the two [Figure 1].

In the classical model of GPCR functionality, ligand binding results in a conformational change of the transmembrane domains, causing it to couple to its heterotrimeric G protein, initiating Gα dissociation from its Gβγ subunits^[27]^. The heterotrimeric G proteins serve as transducers and scaffolding proteins that coordinate downstream signaling. In humans, 16 Gα, 5 Gβ, and 13 Gγ subunits have been identified, leading to a multitude of heterotrimeric combinations^[27]^. The Gα subunit may transduce signals independently of the other subunits, while the Gβγ subunits only signal as a single unit. Gα can be grouped into four major families: α_s_, α_i_, α_q/11_, and α_12/13_. Each of these interacts with different effector proteins, producing second messenger molecules that specifically activate different downstream signaling pathways. The Gα_s_ and Gα_i_ proteins can modulate the effector adenyl cyclase (AC) and subsequently the cAMP levels by stimulating (s) and inhibiting (i) AC, respectively^[28,29]^. The Gα_q/11_ proteins are involved in Ca^2+^ signaling through phospholipase C activation^[30]^. Lastly, Gα_12/13_ proteins are involved in activating small GTP-binding proteins^[31]^. Besides the canonical ligand-activated signaling, some GPCRs display constitutive activity, which is frequently associated with disease development, including cancer^[32]^. In these settings, overexpression of GPCRs is often apparent, which is most often associated with increased basal signaling.

To control GPCR-mediated signaling, negative feedback loops are in place to maintain biological homeostasis. Receptor desensitization takes place upon receptor phosphorylation by G protein-coupled receptor kinases, which allows for β-arrestin recruitment and subsequent internalization of the GPCR, downregulating signaling from the plasma membrane^[22]^. Once the GPCR is in the endosomal system, it can either be recycled back to the plasma membrane or targeted for lysosomal degradation. However, recently, it has become apparent that some GPCRs can also signal from their endosomal compartments upon internalization^[33]^. This changes the spatial-temporal profile of G protein signaling, generating a “second wave” of signaling [Figure 1.4]. This “second wave” of signaling can diverge from the conventionally described GPCR signaling mechanisms, potentially controlling unique physiological as well as pathological downstream effectors. However, such non-canonical signaling is outside the scope of the present review and has been discussed in detail elsewhere^[34]^.

Aside from regulating many physiological processes, dysregulated GPCR expression and/or signaling has been linked to several hallmarks of cancer (reviewed in^[35]^), including proliferation and survival^[36]^, invasion and metastasis^[37]^, angiogenesis^[38]^, and immune cell evasion^[39]^. For instance, in breast cancer, the chemokine receptor CXCR4 is overexpressed and its natural ligand, CXCL12, was shown to be highly secreted near the organs that are the metastatic destination of the tumor cells, indicative of a key role in metastatic colonization^[37]^. Besides the CXCR4/CXCL12 axis, different chemokine receptors have also been shown to play a role in cancer^[37,40-43]^. Other examples of GPCRs involved in cancer progression are the sphingosine 1 phosphate receptors (S1PRs). In glioblastoma, sphingosine-1-phosphate (S1P), the natural ligand for the S1P receptors, stimulates tumor growth and invasiveness^[36]^. S1P stimulates cell proliferation of glioma cells by activating the S1PR_1_ and S1PR_3_ receptors that promote ERK signaling. Notably, the S1PR_2_ receptor appears to have the opposite effect, downregulating ERK activation, suggesting that the S1P-controlled GPCRs may confer contextual mitogenic regulation. Moreover, it was recently demonstrated that other GPCRs affect S1P signaling in glioma development^[39]^. The human cytomegalovirus (HCMV) encoded viral GPCR US28, linked to oncomodulation in several cancers, colocalizes with the S1P_1_ receptor (S1PR1) and recruits the sphingosine kinase 1 (SK1)^[39,44]^. The consequent SK1/S1PR1 signaling stimulates glioma proliferation and survival via AKT, cMYC, and STAT3 pathways. In addition to class A GPCRs, other receptor classes have been linked to cancer progression, including the adhesion^[45,46]^ and metabotropic family^[47,48]^. In comparison to other clinical contexts such as heart disease, brain disorders, and allergies, the use of GPCR targeting agents lags in oncology, where efforts to develop therapeutics have traditionally focused on the smaller family of receptor tyrosine kinases. In the ever-present need for better treatment options for cancer patients, targeting GPCRs may provide unique novel avenues for therapeutic intervention.

## GPCR AND EV INTERPLAY CONTRIBUTES TO ONCOMODULATION

In the last decade, it has become apparent that GPCRs also play roles in several aspects of EV biology, including biogenesis^[49,50]^, sorting of cargo^[51-53]^, secretion^[54]^, and uptake by recipient cells^[55,56]^. In recent years, there has been an increase in studies that link the two in cancer contexts. In this review, we focus on the interplay between GPCRs and EVs in cancer, but that does not exclude that the EV-GPCR cooperation may be relevant for other pathologies as well; for a detailed convergence of EV and GPCR biology, readers are referred to our review^[26]^. GPCRs expressed in a cancerous donor cell may modulate EV secretion or alter EV cargo selection, promoting tumorigenesis when released into the extracellular environment. Alternatively, tumor-derived EVs may deliver molecular components that modulate GPCR signaling when taken up by recipient cells. EVs can also act as carriers for the receptor, horizontally transferring the GPCR from one cell to another, widening the receptor’s potential for modulating oncogenic signaling.

### GPCRs modulate oncogenic EV biogenesis and secretion

**Figure 2:**
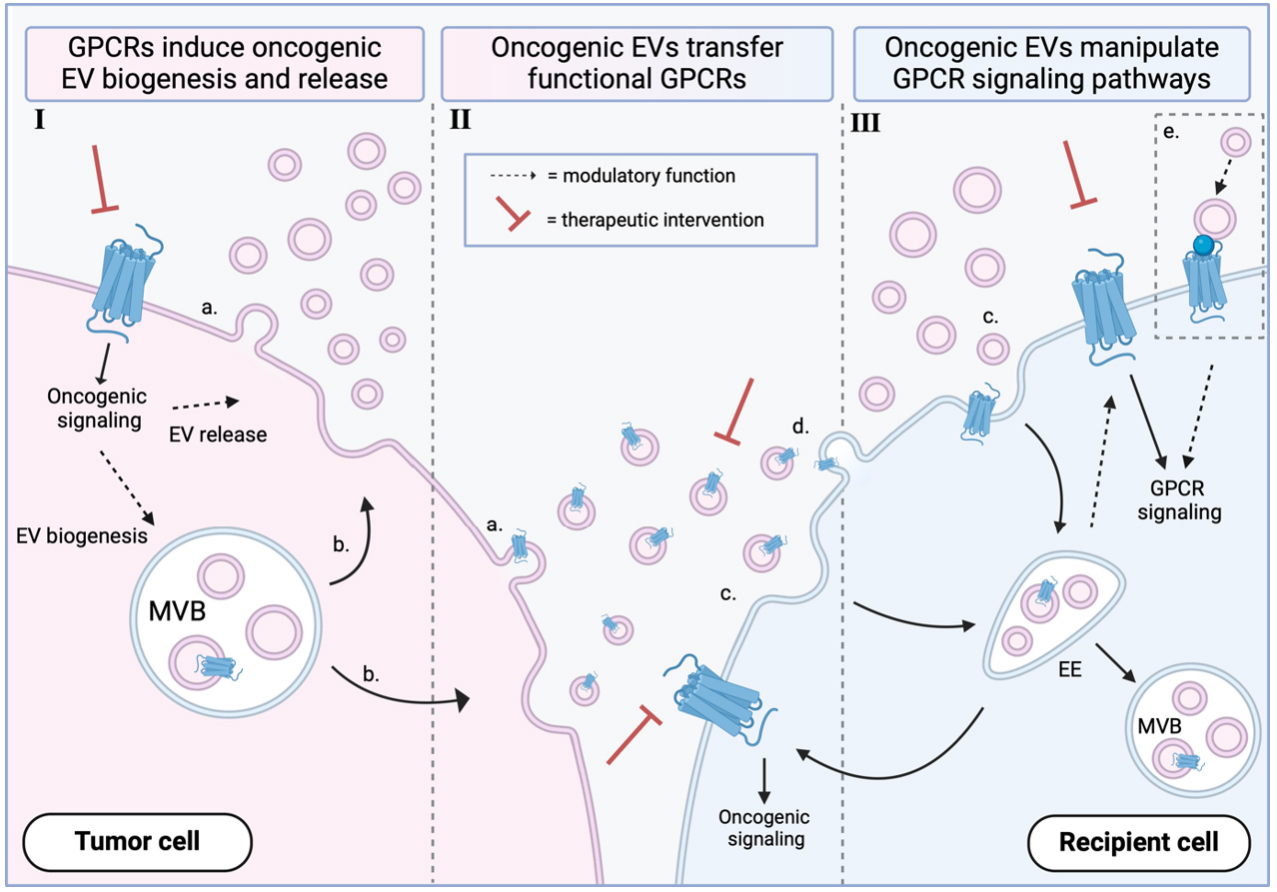
Interplay between GPCRs and EVs during cancer development, highlighting their therapeutic potential. (I) Mutated or oncogenic GPCRs mediate abnormal signaling pathways that can affect protein sorting, leading to a change in EV cargo, or modulate tumor EV biogenesis and secretion, or a combination of the two. (II) EV-mediated functional horizontal transfer of GPCRs can propagate oncogenic signaling in the recipient cells. (III) Uptake of oncogenic EVs, either via endocytosis (c) or perhaps direct membrane fusion (d), can affect endogenously expressed GPCR signaling, contributing to cancer development. Additionally, GPCRs can modulate EV uptake, for example, via docking to the GPCR via its ligand (e). Thus, GPCRs pose an interesting point of interception to target tumor EV-mediated cancer development and progression. a: microvesicle secretion; b: exosome secretion; c: EV endocytosis; d: EV-plasma membrane fusion; e: GPCR-mediated EV docking. GPCR: G protein-coupled receptor; MVB: multivesicular body; EV: extracellular vesicle; EE: early endosome.

Most, if not all, mammalian cells actively release EVs, a family of heterogeneous membrane-enclosed vesicles containing bioactive cargo. Generally, EVs can roughly be categorized into small EVs, when the particles have a diameter < 200 nm, and large EVs, when the particles have a diameter > 200 nm. When the mechanism of EV biogenesis is known, one can also make a distinction between exosomes and microvesicles, two subpopulations of small EVs^[57]^. Microvesicles originate via outward budding of the plasma membrane [Figure 2.I.a], while exosomes evolve from inward budding of the endosomal membrane, generating a multivesicular body containing intraluminal vesicles [Figure 2.I.b]. These vesicles will be recognized as exosomes upon release into the extracellular environment. Due to the overlap in characteristics between EV populations, it is technically challenging to distinguish them unless there is strong evidence on the exact route of biogenesis. Therefore, throughout this review, the general term EV has been chosen in the absence of robust experimental differentiation.

GPCR-mediated signaling has been linked to numerous aspects of EV biology. In fact, several studies describe GPCR-regulated tumor EV secretion [Table 1]. For example, the metabotropic glutamate receptor 3 (mGlu3) has been identified as a driver of EV secretion in breast cancer^[58]^. The activation of mGlu3 in MDA-MB-231 cells resulted in a Rab27a-dependent increased secretion of EVs, while silencing of Rab27a or inhibition of receptor signaling reduced EV release. Furthermore, these tumor EVs appear to carry mitochondrial DNA derived from the MDA-MB-231 cells, which promotes a pro-invasive behavior in recipient cells^[58]^. Similar results were obtained for the bombesin receptor subtype-3 (BRS-3), a GPCR highly expressed in various tumors, such as breast cancer, lung cancer, and prostate cancer^[59,60]^. Activation of BRS-3 in stably expressing HEK293 cells leads to a 50% increase in secretion of EVs compared to the unstimulated condition, while antagonist treatment reversed these effects, hinting at BRS-3 dependency^[59]^. Interestingly, BRS-3 itself was shown to be sorted into these EVs and horizontally transferred to recipient cells. Stimulation of the GPCR in the recipient cells resulted in elevated ERK phosphorylation, suggesting that BRS-3 retained its function upon transfer. These observations suggest that GPCRs can be responsible for the secretion of tumor-derived EVs as well as for their cargo composition including the horizontal transfer of the GPCRs themselves.

**Table 1:** Overview of GPCRs involved in EV-mediated cancer progression

**GPCR location**	**GPCR involved**	**Cancer type**	**Oncogenic effect**	**Ref.**
**Class A**				
On EV-secreting cell	PAR2 (protease-activated receptor)	Breast cancer	PAR2 activation stimulates EV shedding from the plasma membrane. This process is regulated by MAPK, p38, and Rho signaling. These EVs also incorporate components that drive the invasion and migration of recipient cells	[76]
	H1R1 (histamine receptor)	Cervical cancer	Activation of H1R1 induces SNAP23-dependent fusion of the multivesicular body with the plasma membrane, resulting in increased EV secretion	[77]
Incorporated into EVs	CXCR4 (chemokine receptor)	Murine hepatocarcinoma	Horizontal transfer of CXCR4 via EVs from cells with high metastatic potential increases the motility of cells with a low metastatic potential	[62]
	CXCR4	Acute myeloid leukemia (AML)	AML cells secrete CXCR4-positive EVs that can be horizontally transferred to leukemia cells lacking CXCR4, increasing metastatic spread, growth, and bone marrow infiltration via the CXCL12-CXCR4 axis	[63]
	CXCR4	Glioblastoma	Glioblastoma-derived EVs carry CXCR4 monomers and dimers, as well as VEGF. Treatment of endothelial cells with these EVs promotes cell proliferation, motility and tube formation compared to control EVs	[78]
	CXCR4	Breast cancer	A liver-kidney-on-a-chip model shows breast cancer-derived EVs organ tropism. MCF7 and MDA-MB-231 cells secrete CXCR4^+^ EVs that drive liver-kidney tropism via an CXCL12 dependent gradient	[79]
	US28 (viral chemokine receptor)	Glioblastoma	US28-positive EVs retain binding to chemokines in the extracellular environment. Further, the US28-EVs attenuate CX_3_CL1-CX_3_CR1 signaling in EV-treated HEK293T cells	[66]
	S1P2 (sphingolipid receptor)	Breast cancer	Breast cancer cell-derived EVs horizontally transfer an N-terminally processed form of S1P2 to fibroblast, where it constitutively activates ERK signaling	[64]
	CCR6, CX_3_CR1 (chemokine receptors)	Lung, pancreatic and colorectal cancer	Tumor EVs shed by various cancer cells carry tumor cell markers CCR6 and CX_3_CR1. CCR6 was transferred to monocytes, promoting chemotaxis	[80]
	CCR1, CCR6, CXCR4 (chemokine receptors)	Gastric cancer	The expression of chemokine receptors on EVs from gastric cancer patients seems to vary depending on the cancer stage (i.e., CCR6 expression goes up while CXCR4 expression goes down)	[81]
On EV-receiving cell	CXCR2, CXCR4 (chemokine receptors)	Melanoma	EV-mediated skewing of neutrophils toward a tumor-promoting phenotype, yielding tumor cell survival and migration	[70]
	ACKR3, CXCR4 (chemokine receptors)	Melanoma	Increased migration of non-osteotropic melanoma cells due to EV-mediated upregulated expression of ACKR3	[71]
	CXCR4	Prostate cancer	EVs mediate the transfer of PKM2 to bone marrow cells, inducing CXCL12 secretion and subsequent CXCR4-dependent migration of the cancerous cells to the bone marrow	[72]
	CXCR4	Breast cancer	Platelets-derived microvesicles increase the expression of CXCR4 in recipient breast cancer cells, partially enhancing their chemo invasiveness	[82]
	ACKR3	Colorectal cancer	ACKR3-overexpressing tumor cells secrete EVs containing miR146a-5p and miR155-5p. These microRNAs are endocytosed by cancer-associated fibroblasts which get activated via JAK2-STAT3/NF-B-dependent signaling. Subsequently, these fibroblasts secrete inflammatory cytokines, including CXCL12. CXCL12 activates ACKR3 on the tumor cells, inducing EMT and pre-metastatic niche formation	[83]
	CCR8 (chemokine receptor)	Glioblastoma, malignant melanoma and lung carcinoma	CCR8 facilitates the uptake of EVs carrying TMZ resistance	[55]
	PAR (protease-activated receptor)	Triple-negative breast cancer	EVs rich in MMP1 from TNBC cells with a high metastatic potential promote metastasis of low metastatic cells via PAR1-mediated EMT	[73]
	S1PR1, S1PR2 (sphingolipid receptor)	Ovarian cancer	Transfer of tumor-derived EVs rich in SPHK1 increases the S1P production, leading to S1PR1/2-dependent PD-L1 expression	[74]
	A_2B_ (adenosine receptor)	Head and neck squamous cell carcinoma	Tumor EVs, rich in adenosine, promote the secretion of angiogenic factors via adenosine A_2B_ signaling	[84]
	CCR2 (chemokine receptor)	Breast cancer	Tumor EVs, decorated with CCL2, migrate and accumulate near cells expressing CCR2, driving metastatic spread	[56]
	A_2A_ (adenosine receptor)	Bladder, colorectal, prostate and breast cancer	Tumor EVs carrying CD39 and CD73 covert ATP into adenosine, which activates adenosine A2A signaling in immune cells, regulating an immune response	[85]
	EP2, EP4 (prostanoid receptors)	Prostate cancer	Tumor-derived EVs, rich in PGE2, induce the expression of CD73 on dendritic cells. CD73 increases the extracellular levels of adenosine, which inhibits T cell functioning	[86]
	CX_3_CR1 (chemokine receptor)	Prostate cancer	Tumor EV-treated fibroblast release EVs carrying membrane-bound CX_3_CL1 that promotes migration and invasion of cancer cells via the CX_3_CL1-CX_3_CR1 signaling axis	[87]
	LPAR1, LPAR3 (lysophosphatidic acid receptors)	Not specified	ATX bound EVs can sequester LPA and activated LPAR1/3 signaling. LPARs are often involved in tumor stroma interactions and metastasis	[88,89]
	PAR1	Breast and pancreatic cancer	Tumor EVs carry activated factor X on the EVs through which they can activate PAR1 receptors, promoting metastasis and pre-metastatic niche formation	[90]
**Class B**				
On EV-secreting cell	CD97/ADGRE5 (adhesion receptor)	Gastric cancer	CD97 expression ensures incorporation of EV cargo that promotes tumor cell proliferation and invasion via MAPK signaling or increases metastatic effects	[91,92]
Incorporated into EVs	ELTD1/ADGRL4 (adhesion receptor)	Breast cancer	Adhesion receptor ELTD1/ADGRL4, and its highly glycosylated form, are incorporated into EVs and horizontally transferred to endothelial cells, promoting endothelial sprouting	[65]
**Class C**				
On EV-secreting cell	mGLU3 (metabotropic glutamate receptor)	Breast cancer	Receptor activation results in a Rab27-dependent increase in tumor EV secretion, as well as incorporation of specific cargo that increases invasive behavior of the recipient cells	[58]
	GRM1 (metabotropic glutamate receptor)	Melanoma	Metabotropic glutamate receptor 1 GRM1 activation promotes the incorporation of cargo that modulates cell migration, invasion, and growth of GRM1-negative recipient cells	[51]
Incorporated into EVs	GPRC5C (metabotropic glutamate receptor)	Pancreatic cancer	GPRC5C is incorporated into EVs of cancer patients but not of healthy subjects, potentially serving as a biomarker. Note: in breast cancer, knockdown of GPRC5C promotes cell proliferation	[93,94]
**Class F**				
Incorporated into EVs	FZD-10 (frizzled receptor)	Colorectal and gastric cancer, hepatocarcinoma, cholangiocarcinoma	FZD-10 protein incorporation into EVs is upregulated in cancer patients compared to healthy subjects. EVs carrying FZD-10 as cargo increase tumor cell proliferation and metastasis	[95,96]
On EV-receiving cell	FZD (frizzled receptor)	Breast cancer	Tyrosine kinases ROR1 and ROR2 are being transferred via EVs to ROR1/2-negative cancer cells, acting as co-receptors for the Wnt signaling pathway, driving cancer progression	[97]
	FZD-6 (frizzled receptor)	Breast cancer	Cancer-associated fibroblast EVs get processed by breast cancer cells, promoting attachment of autocrine Wnt11. These EVs in turn promote the protrusive activity, motility and metastasis of the breast cancer cells	[98]
	FZD (frizzled receptor)	Colorectal cancer	Cancer-associated fibroblast EVs rich in Wnt molecules dedifferentiate colorectal cancer cells via Wnt-FZD-dependent signaling, conferring chemotherapy resistance	[99]
**Orphan GPCRs**				
On EV-secreting cell	GPR143/OA1 (ocular albinism type 1 receptor)	Melanoma, breast, colorectal cancer	GPR143 regulates the ESCRT machinery, thereby determining the EV content and quantity. In mice, EVs secreted from GPR143-positive cells increased the migratory and invasive potentials	[61]
On EV-secreting cell and incorporated into EVs	BRS-3 (bombesin-like receptor)	Lung, breast, and prostate cancer	BRS-3 activation yields a 50% increase in EV secretion, which is Rho signaling-dependent. Further, BRS-3 was incorporated into the EVs and functionally transferred to recipient cells	[59,60]

As briefly discussed above, GPCRs can also influence the composition of tumor EVs. Recently, the GPCR GPR143 was identified as a regulator of “endosomal sorting complex required for transport” (ESCRT)-dependent EV biogenesis^[61]^. Knockdown of GPR143 in MCF7 cells reduces the amount and alters the protein content of EVs. The downregulated proteins are involved in cell motility and invasion pathways. In addition, exposure of MCF7 cells to EVs derived from GPR143-overexpressing breast cancer cells (MCF-7) enhanced their migration and invasion compared to EVs from shRNA against GPR143 containing cells, while exposure of HUVEC cells to the former EVs promoted their vascular branching^[61]^. Interestingly, the ability of GPR143-dependent EVs to modulate cell motility was linked to specific integrins. This finding indicates that GPR143-mediated signaling may be responsible for the selection of the EV cargo. Comparable results were obtained for the metabotropic glutamate receptor 1 (GRM1)^[51]^. Expression of this GPCR in C81-61 melanoma cells does not increase the secretion of EVs; however, it does change the composition of these EVs^[51]^. C81-61 GRM-cells treated with EVs derived from C81-61 cells expressing GRM1 displayed an increase in migration and invasion, suggesting a role in cargo selection.

Taken together, these studies underscore that GPCRs form a potential therapeutic target when located on the EV-secreting cells themselves. Modulation of these receptors and/or their downstream signaling pathways may downregulate EV secretion or affect oncogenic cargo selection, attenuating cancer progression [Figure 2.I].

### Tumor EVs incorporate and transfer functional GPCRs to recipient cells

*Tumor cell migration and invasion* - In addition to regulating EV secretion and composition, GPCRs can be included as molecular cargo within EVs [Table 1, Figure 2.II]. The metastatic potential of a tumor cell can be altered upon importing particular receptors, as they may affect whether and how the cell can then respond to extracellular stimuli. For instance, exposure to EVs isolated from mouse hepatocarcinoma cells with a high metastatic potential (Hca-F cells) increased the migratory and invasive capacity of hepatocarcinoma cells with low metastatic behavior (Hca-P cells)^[62]^. Transfer of chemokine receptor CXCR4 via EVs seemed particularly important in the EV-mediated transition to a more metastatic profile observed in Hca-P cells. These modified Hca-P cells significantly increased the expression of CXCR4. In line with this, CXCR4 knockdown in Hca-F cells yielded a lower level of CXCR4 in Hca-P cells and the same observation could be made upon inhibition of EV transfer, suggesting CXCR4 being trafficked between cells via EVs. Another study demonstrated that EVs are involved in acute myelogenous leukemia (AML)^[63]^. The EVs isolated from peripheral blood and bone marrow plasma were shown to have elevated protein levels of CXCR4 compared to samples of healthy individuals. Upon exposure of HL-60 cells to these EVs, CXCR4 levels significantly increased, as did the migratory potential toward CXCL12, suggesting EV-mediated transfer of a functional CXCR4 receptor.

*Components undergo a contextual shift -* Besides the direct transfer and uptake of functional GPCRs into recipient cells, some receptors undergo alterations during this process. This contextual switch could cause the GPCR to change conformation, specific G protein coupling, or downstream altered coupling to effector proteins. In breast cancer, the S1P_2_ receptor has been implicated in the stimulation of fibroblast cell proliferation via an EV-dependent manner^[64]^. MDA-MB-231 cell-derived EVs contain S1P_2_ receptors that are transferred to fibroblasts. Treatment of MEF fibroblasts with media S1P_2_-EV containing resulted in ERK1/2 activation and DNA synthesis. Interestingly, activating the endogenous S1P_2_ receptor with allosteric agonist CYM5520 on the recipient cells did not yield an increase in DNA synthesis according to the [^3^H]-thymidine incorporation assay. In addition, inhibition of S1P binding to S1P_2_ and subsequent receptor activation did not affect ERK1/2 activation in the EV-treated MEF cells, suggesting that the receptor obtained constitutive activity. These findings were validated by S1P_2_ knockdown in the donor cells, whose EVs failed to activate ERK1/2 in the fibroblasts. The transferred S1P_2_ receptor appeared to be structurally altered; upon uptake, it gets processed to a shorter form due to cleavage of the N-terminal domain, which results in a GPCR conformation that constitutively enables ERK1/2 activation. The consequences of proteolytic activation of this receptor in cancer progression have yet to be determined. However, it is tempting to speculate that increased fibroblast proliferation might result in myofibroblast transitioning, stromal cells known to be involved in cancer progression. Similarly, the adhesion GPCR, ADGLR4, formerly referred to as ELTD1, was recently identified in EVs^[65]^. This receptor is involved in vasculature formation and resistance to anti-angiogenic therapy. It has been demonstrated that ADGRL4/ELTD1 is taken up in EVs from HUVEC and HMEC cells mostly in its highly N-glycosylated form (ECD hyperglycosylated). Detachment of the hyperglycosylated ECD results in receptor activation, suggesting that the EVs may carry specifically the activated form of the GPCR. Upon exposure of healthy HUVEC cells with these ADGRL4/ELTD1-containing EVs, cell proliferation and endothelial sprouting significantly increased both *in vitro* and *in vivo*^[65]^. This suggests that ADGRL4/ELTD1 incorporation into EVs might increase the activity of the receptor and have a functional consequence on angiogenesis when transferred to endothelial cells.

*Implications on viral pathogenesis -* EVs have not only been implicated in the incorporation of endogenous GPCRs. Infecting pathogens like viruses can also encode GPCRs that can be expressed in tumorigenic cells, such as the HCMV-encoded US28 - a viral GPCR that has oncomodulatory properties in several cancers^[44]^. Recently, we discovered that this receptor is sorted into EVs secreted from U251 glioma cells^[66]^. Over the course of host-virus co-evolution, HCMV acquired the human chemokine C-X_3_-C motif receptor 1 (CX_3_CR1), which has since diverged into US28, displaying structural homology and allowing it to bind a broad spectrum of human chemokines^[67]^. Interestingly, US28-positive EVs retain their ability to bind these chemokines^[66]^. Context was provided by experiments showing that US28^+^ EVs derived from U251 cells could scavenge away chemokines from the human CX_3_CR1, reducing receptor activation in HEK293T cells. One could speculate that this could lead to a suppressed immune reaction by attenuating immune cell migration toward the site of infection. If, indeed, chemokine receptor-decorated EVs were shown to disrupt chemoattractant gradients sufficient to deflect immune cell patrol, this spurs curiosity about the spread of such a mechanism in other (patho)physiological contexts. One could imagine that in a cancer setting, for example, tumor cells (beyond those HMCV-infected) would benefit from a receptor-decorated EV “sponge” to control local chemoattractant gradients and hence an immune cell invitation list within the tumor microenvironment. EV-mediated horizontal transfer of US28 has yet to be investigated; however, such a process cannot be excluded as a potential way for viral spread or pre-metastatic niche formation. In fact, EV-mediated transfer of CXCR4^[68]^ and CCR5^[69]^ to increase viral spread has already been demonstrated in the human immunodeficiency virus.

### Tumor-derived EVs mediate oncogenic GPCR signaling in recipient cells

GPCRs are not just interesting therapeutic targets as EV cargo molecules. EVs could also act as carriers of a host of ligands, co-receptors, or other molecules that go on to activate GPCRs, thereby eliciting oncogenic signal transduction in receiving cells. A considerable number of studies have indicated that tumor-specific EV cargo may influence GPCR signaling in recipient cells [Table 1, Figure 2.III].

**Figure 3:**
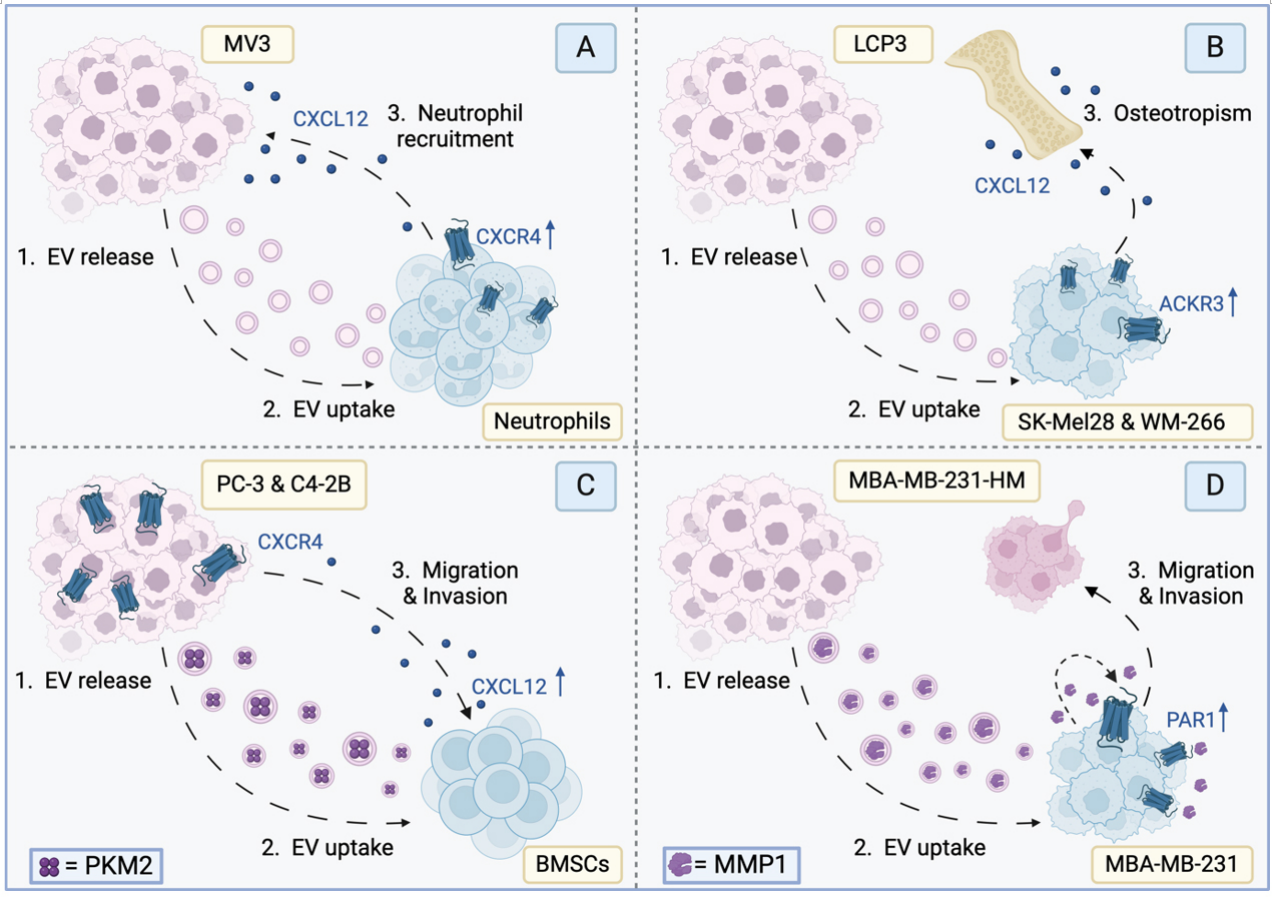
Illustrative examples of EV-GPCR oncomodulation. (A) MV3 melanoma cell-derived EVs activate primary neutrophils to adopt a pro-tumor/N2-phenotype by increasing the expression of chemokine receptor CXCR4. This allows the neutrophils to migrate toward the tumor cells in a CXCL12-dependent manner, promoting tumor cell survival; (B) Osteopathic melanoma LCP-derived EVs alter the osteotropism of other melanoma cells. EV exposure of SK-Mel28 and WM-266 cells induces plasma membrane ACKR3 expression, resulting in a CXCL12-dependent tumor cell migration toward the bone; (C) EVs derived from prostate cancer cells are rich in PKM2, a kinase that induces CXCL12 production and secretion in bone marrow stem cells. Increased CXCL12 section induces migration of cancer cells toward the bone marrow in a chemokine receptor CXCR4-dependent manner; (D) Breast cancer cells can increase the migration and invasion of other breast cancer cells via EV-mediated transfer of MMP1. MMP1 is a protease that can activate PAR1 receptors on the receiving breast cancer cells in an autocrine manner, resulting in increased migration. PKM2: pyruvate kinase muscle isozyme 2; MMP1: matrix metalloprotease 1; BMSCs: bone marrow stem cells.

*Pre-metastatic niche formation* - EVs have been shown to be important components in “tumor-education” of immune cells. A recent study demonstrated that MV3 melanoma-derived EVs can activate primary human neutrophils to adopt a pro-tumor/N2 phenotype, driving tumor progression [Figure 3A]^[70]^. The tumor-released EVs induce neutrophil migration toward the tumor microenvironment through the chemokine receptor CXCR2/CXCL8 (IL8) axis, activating the PI3K-AKT pathway in the neutrophils. Activation of this signaling pathway resulted in an increased transcriptional level of CXCR4 in the EV-exposed neutrophils, which was abrogated upon treatment with the PI3K inhibitor LY294002. Furthermore, co-cultured EV-treated neutrophils yielded an increase in tumor cell viability compared to MV3 cells co-cultured with naïve neutrophils. Taken together, these data suggest that tumor-derived EVs may increase the transcriptional level of CXCR4 in neutrophils, skewing their phenotype to a pro-tumor polarization, allowing tumor cell survival.

Bone metastases rarely occur in patients suffering from malignant melanoma. However, when they do, it severely worsens prognosis. The effects of melanoma-derived EVs on the propensity of non-osteotropic cells to be attracted toward bone (osteotropism) have been investigated. EVs derived from osteotropic LCP melanoma cells appear to stimulate the osteotropic behavior of SK-Mel28 and WM-266, non-osteotropic tumor cells, toward bone in a CXCL12-stimulated manner through the atypical chemokine receptor 3 (ACKR3)/CXCR4 axis [Figure 3B]^[71]^. Exposure to LCP-derived EVs upregulates the level of plasma membrane-localized ACKR3 in SK-Mel28 and WM-266 cells. In contrast, plasma membrane levels of CXCR4, also activated by CXCL12, are not affected after exposure to LCP-derived EVs. The tumor-derived EVs did not carry CXCR4 nor ACKR3 protein, suggesting the transfer of other molecules that induce ACKR3 expression. However, silencing of either of the GPCRs attenuates the migratory properties these non-osteotropic cells obtain after EV treatment. This proves that the gained osteotropism of SK-Mel28 and WM-266 cells is driven by the CXCL12/CXCR4/ACKR3 axis, which appears to be stimulated in the recipient cells through EV-mediated upregulation of plasma membrane ACKR3. Similar results were reported in the context of prostate cancer, where tumor EVs promote pre-metastatic niche formation, enhancing bone metastasis^[72]^. In this case, prostate cancer PC-3 and C4-2B cell-derived EVs are rich in pyruvate kinase muscle isozyme M2 (PKM2) and transfer this protein to bone marrow stem cells (BMSCs). In BMSCs, the increase in protein levels of PKM2 upregulates CXCL12 production in a HIF-1α-dependent manner^[72]^. This attracts the prostatic cancer cells through CXCR4 and enhances their growth in bone marrow [Figure 3C]. CXCL12 is a clinically targetable protein, but blocking this chemokine comes with various immune-related adverse events. Thus, targeting the GPCR involved could be a reasonable approach to counter the EV-mediated metastasis.

*EVs in establishing a tumor microenvironment -* Apart from tumor-stromal crosstalk, EV-mediated communication can also occur between the tumor cells themselves. In triple-negative breast cancer (TNBC), tumor EVs have been shown to enhance the metastatic potential of other TNBC cells^[73]^. MDA-MB-231-HM cells with high metastatic potential secrete EVs enriched in matrix metalloprotease 1 (MMP-1). MMP-1 activates PAR1, a protease-activated GPCR involved in migration and invasion, on the recipient MDA-MB-231 cells by cleaving the N-terminal domain. It was hypothesized that MMP1 is packed into the EVs of the donor cell and transferred to the receiving cells. These cells then release the protease into the extracellular environment, so it can activate PAR1 in an autocrine manner, promoting migration and invasion of TNBC [Figure 3D]^[73]^. Another interesting study demonstrated that ovarian cancer-derived EVs are packed with SPHK1/SK1, which catalyzes the phosphorylation of S1P^[74]^. S1P was shown to elevate programmed cell death ligand 1 (PD-L1) expression on three different ovarian cell lines - OVCAR5, HeyA8, and OVCAR4, driving T cell exhaustion. Moreover, it was demonstrated that silencing S1PR1 and S1PR2, two GPCRs that are activated via S1P binding, yielded a significant reduction in PD-L1 expression in OVACR5 cells^[74]^. One might hypothesize that EVs transfer SPHK1/SK1 to cells in the tumor microenvironment, elevating their production of S1P. In turn, S1P could activate its receptors on the recipient cells, leading to the reported PD-L1 overexpression and subsequently immune evasion.

*GPCRs as cellular anchors -* Modulation of GPCR signaling pathways within the recipient cells is not the only mode of EV-mediated action. GPCRs can also be used as docking sites, connecting EVs with the recipient cells. In glioblastoma, it was shown that the chemokine receptor CCR8 acts as a docking site for tumor-derived EVs^[55]^. CCR8 binds to the soluble ligand CCL18, which in turn binds to glycans on the EVs; thereby, CCL18 acts as a bridging molecule between the cells and the EVs. A “chemokine cloud” model where glycans cause chemokine retention near the EVs by constantly engaging with them, trafficking the EVs toward cells expressing the chemokine receptor, was postulated as the mode of action^[55,75]^. This study shows CCR8’s involvement in the EV-mediated transfer of chemoresistance-determining factors between glioblastoma cells^[55]^. CCR8 appears to ensure EV uptake and cargo transfer, enhancing cell growth and protecting the cells against temozolomide, a chemotherapeutic. The latter was counteracted by blocking CCR8, re-sensitizing the recipient GBM8 cells to temozolomide *in vitro* and *in vivo*. These are promising findings that strengthen the notion of targeting GPCRs as a therapeutic approach to counteract tumor EV-mediated effects [Figure 2.III.e].

## CLINICAL APPLICATIONS

The field of EV therapeutic applications is experiencing significant development. Pre-clinical data suggest that EVs hold promise in many aspects of biomedicine, ranging from diagnostic monitoring to active treatment. Traditionally, cancer diagnosis involves invasive biopsy procedures, whereas clinically informative circulating EVs can be obtained via liquid biopsies, such as urine or blood. By carrying GPCRs and/or their ligands, EVs could open new avenues as biomarkers for cancer diagnosis or treatment monitoring. For example, in the case where EVs obtained from blood and plasma samples of AML patients showed increased levels of protein CXCR4 and CXCL12 compared to EVs from healthy control samples^[63]^. Similarly, EVs derived from breast and prostate cancer cells are packed with MMP1 and PKM2, respectively, compared to EVs derived from their healthy counterparts^[72,73]^. EVs are also currently being explored as drug delivery systems due to their many biocompatible features. In comparison to conventional drug delivery systems, EVs might overcome the hurdle of cytotoxicity and immunogenicity due to their natural origins. One could, therefore, envisage that EVs loaded with drugs targeting oncogenic GPCR signaling hubs may elicit an antitumor response. Many hurdles still need to be overcome, including EV production and standardization at scale, or large clinical trials for biomarker incorporation into treatment regimes. In parallel, given the wide range of GPCR physiological functions, any potential EV-GPCR targeting strategy must be rigorously tested to determine whether a sufficient therapeutic window exists. Nevertheless, the field holds excitement for the promise of EVs in next-generation therapeutics, from which GPCR and EV researchers may benefit.

## CONCLUDING REMARKS

Reports of marked upregulation of EV release from cancer cells catapulted research interest in the field. Evidence of the plethora of cancer-supporting roles continues to accumulate - positioning EV-mediated communication as the target of therapeutic interjection. Yet, being a diverse and malleable communication channel, the right choice of molecular target remains elusive. In parallel, pharmacological research in the last decades has heralded the GPCR family as their muse: not only becoming the largest family of approved drug targets, but also the system by which many new pharmacological strategies are probed and developed. And crucially, where many paradigm shifts in our fundamental understanding of cellular signaling have been discovered - with repercussions far beyond the receptors themselves. While estimates vary on both the number of GPCRs and the number of drugs that target these, what is clear is the unique therapeutic potential housed within this family of proteins. Numerous factors underlie this - the vast number of receptors within the family, their structural druggability, interaction with key cellular mediators, and their presence in the plasma membrane. To therapeutically intercept oncogenic EV-mediated communication, a *rational* and *actionable* molecular target that regulates key steps within bidirectional onco-EV channels is required. The studies collated herein demonstrate that, ultimately, there may be therapeutic benefits in cross-targeting EVs through GPCRs, as they represent nodes at crucial points of the EV life cycle in various oncological settings.

## DECLARATIONS

### Copyright

© The Author(s) 2024.
